# Supplementary material for: A preliminary cost-utility analysis of routine myasthenia gravis and thyroid dysfunction screening in acquired comitant Esotropia
Source: PLoS One. 2026 May 28;21(5):e0350280. doi: 10.1371/journal.pone.0350280 (PMC13218454; doi:10.1371/journal.pone.0350280)
Supplement: S1 Table — This table details presenting features, laboratory and imaging findings, final diagnoses, and early treatment outcomes for patients with abnormal AChR-Ab or TFT results. Abbreviations: CT, computed tomography; MRI, magnetic resonance imaging; MG, myasthenia gravis; OMG, ocular myasthenia gravis; F, female; TSH, thyroid-stimulating hormone; FT3, free triiodothyronine; FT4, free thyroxine. (DOCX) [file pone.0350280.s004.docx]

**S1 Table. Clinical and Diagnostic Characteristics of Patients with Positive Laboratory Findings**

| **Patient** | **Age/Sex** | **Presenting Symptoms** | **Positive Lab Results** | **Imaging** | **Confirmed Diagnosis** | **Treatment** | **Initial Outcomes** |
| --- | --- | --- | --- | --- | --- | --- | --- |
| OMG #1 | 28/F | Horizontal diplopia (1 yr) | AChR-Ab 6.27 nmol/L | Normal | OMG | Pyridostigmine 60 mg/day | Diplopia resolved |
| OMG #2 | 4/F | Esotropia (3 mo) | AChR-Ab IgG 0.42 (borderline, confirmed by RNS) | Normal | OMG | Pyridostigmine | Squint improved |
| OMG #3 | 68/F | Diplopia (5 yrs) | AChR-Ab 6.92 nmol/L | Normal | OMG | Pyridostigmine 60 mg/day | Improved with prism |
| Hypothyroid | 74/F | Diplopia (4 yrs) | TSH 5.9, FT3 1.06, FT4 2.36 | Normal | Subclinical hypothyroidism | Euthyrox | Improved with prism |

This table details presenting features, laboratory and imaging findings, final diagnoses, and early treatment outcomes for patients with abnormal AChR-Ab or TFT results.

**Abbreviations:** CT, computed tomography; MRI, magnetic resonance imaging; MG, myasthenia gravis; OMG, ocular myasthenia gravis; F, female; TSH, thyroid-stimulating hormone; FT3, free triiodothyronine; FT4, free thyroxine.
