# Supplementary material for: A preliminary cost-utility analysis of routine myasthenia gravis and thyroid dysfunction screening in acquired comitant Esotropia
Source: PLoS One. 2026 May 28;21(5):e0350280. doi: 10.1371/journal.pone.0350280 (PMC13218454; doi:10.1371/journal.pone.0350280)
Supplement: S2 Table — This table summarizes the health utility values applied to estimate quality-adjusted life years (QALYs) across diagnostic and treatment states in the economic model. Abbreviations: MG, myasthenia gravis; OMG, ocular myasthenia gravis; ADL, activities of daily living; TED, thyroid eye disease; QALY, quality-adjusted life year. (DOCX) [file pone.0350280.s005.docx]

**S2 Table. Utility Weights for Myasthenia Gravis (MG), Ocular Myasthenia Gravis (OMG), and Thyroid Disorders**

| **Condition** | **Severity** | **QALY Value** | **Reference** |
| --- | --- | --- | --- |
| MG | Generalized MG | 0.739 | [17] |
|  | Mild MG (MG-ADL 0–4) | 0.872 | [17] |
|  | Moderate MG (MG-ADL 5–9) | 0.707 | [17] |
|  | Severe MG (MG-ADL ≥10) | 0.511 | [17] |
|  | Well-controlled MG / Remission | 0.980 | [17] |
| OMG | Early diagnosis (treated) | 0.872 | [17] |
| Hypothyroidism | Subclinical | 0.950 | [18] |
|  | Overt (treated) | 0.940 | [18] |
| TED | Moderate–severe | 0.440 | [19] |
|  | Severe (constant diplopia, large proptosis) | 0.300 | [19] |
|  | Severe (constant diplopia, small proptosis) | 0.340 | [19] |
|  | Mild (no diplopia, small proptosis) | 0.600 | [19] |

This table summarizes the health utility values applied to estimate quality-adjusted life years (QALYs) across diagnostic and treatment states in the economic model.

**Abbreviations:** MG, myasthenia gravis; OMG, ocular myasthenia gravis; ADL, activities of daily living; TED, thyroid eye disease; QALY, quality-adjusted life year.
