# Supplementary material for: A preliminary cost-utility analysis of routine myasthenia gravis and thyroid dysfunction screening in acquired comitant Esotropia
Source: PLoS One. 2026 May 28;21(5):e0350280. doi: 10.1371/journal.pone.0350280 (PMC13218454; doi:10.1371/journal.pone.0350280)
Supplement: S3 Table — Costs are presented in 2024 Thai Baht (THB), discounted at 3% annually following Thai Health Technology Assessment (HTA) guidelines. Unit costs were obtained from national hospital billing data and adjusted for inflation using the Thai consumer price index (CPI). Quantities reflect observed case prevalence and modeled cohort size (n = 110). Abbreviations: AChR-Ab, acetylcholine receptor antibody; TFT, thyroid function test; TSH, thyroid-stimulating hormone; FT3, free triiodothyronine; FT4, free thyroxine; MRI, magnetic resonance imaging; CT, computed tomography; THB, Thai Baht; TP, true positive; FP, false positive; OMG, ocular myasthenia gravis. (DOCX) [file pone.0350280.s006.docx]

**S3 Table. Cost Components by Diagnostic Strategy (10-Year Horizon, 3% Discount)**

| **Strategy** | **Cost Component** | **Unit Cost (THB)** | **Quantity (per cohort of 110)** | **Total Cost (THB)** |
| --- | --- | --- | --- | --- |
| **Universal Screening** | AChR-Ab test | 1,900 | 110 | 209,000 |
|  | TFT (TSH + FT3 + FT4) | 490 | 110 | 53,900 |
|  | MRI brain/orbit | 18,500 | 110 | 2,035,000 |
|  | CT chest (for thymoma workup) | 8,200 | ~3.3 (TP + FP cases) | 27,224 |
|  | Follow-up visits (6 × 110) | 750 | 660 | 495,000 |
|  | Mestinon (OMG, TP only) | 240/month | 81 months total | 19,440 |
|  | Euthyrox (hypothyroid TP only) | 45/month | 12 months total | 486 |
| **Total (Screening)** |  |  |  | **2,840,050** |
| **No Screening** | MRI brain/orbit | 18,500 | 110 | 2,035,000 |
|  | ICU stay (2 pts × 5 days) | 5,500/day | 10 | 55,000 |
|  | Misdiagnosis-related costs | — | — | 900,000 |
|  | Follow-up visits (same as screening) | 750 | 660 | 495,000 |
| **Total (No Screening)** |  |  |  | **3,485,000** |

Costs are presented in 2024 Thai Baht (THB), discounted at 3% annually following Thai Health Technology Assessment (HTA) guidelines. Unit costs were obtained from national hospital billing data and adjusted for inflation using the Thai consumer price index (CPI). Quantities reflect observed case prevalence and modeled cohort size (n=110).

**Abbreviations:** AChR-Ab, acetylcholine receptor antibody; TFT, thyroid function test; TSH, thyroid-stimulating hormone; FT3, free triiodothyronine; FT4, free thyroxine; MRI, magnetic resonance imaging; CT, computed tomography; THB, Thai Baht; TP, true positive; FP, false positive; OMG, ocular myasthenia gravis.
