# Supplementary material for: A preliminary cost-utility analysis of routine myasthenia gravis and thyroid dysfunction screening in acquired comitant Esotropia
Source: PLoS One. 2026 May 28;21(5):e0350280. doi: 10.1371/journal.pone.0350280 (PMC13218454; doi:10.1371/journal.pone.0350280)
Supplement: S4 Table — This table summarizes the input assumptions for epidemiologic, diagnostic, cost, and utility variables used in the decision-analytic model. Parameter ranges reflect ±20% variation from base-case estimates unless otherwise specified. For probabilistic sensitivity analysis (PSA), beta distributions were assigned to probabilities and utilities, and gamma distributions to cost parameters. Abbreviations: ACE, acquired comitant esotropia; AChR-Ab, acetylcholine receptor antibody; HTA, health technology assessment; MG, myasthenia gravis; OMG, ocular myasthenia gravis; QALY, quality-adjusted life year; TFT, thyroid function test; TSH, thyroid-stimulating hormone; FT3, free triiodothyronine; FT4, free thyroxine; THB, Thai Baht; WTP, willingness-to-pay. (DOCX) [file pone.0350280.s007.docx]

**S4 Table. Model Input Parameters for Cost–Utility Analysis**

| **Parameter** | **Base-Case Value** | **Range / Distribution** | **Source** |
| --- | --- | --- | --- |
| MG prevalence in ACE | 2.7% | 1.5%–5.0% | Study cohort |
| Hypothyroidism prevalence in ACE | 0.9% | 0.5%–2.0% | Study cohort |
| AChR-Ab test sensitivity | 75% | Beta distribution | [6,7] |
| AChR-Ab test specificity | 98% | Beta distribution | [6] |
| TFT sensitivity (TSH-first reflex FT4/FT3) | 90% | Beta distribution | [8–10] |
| TFT specificity | 92% | Beta distribution | [8–10] |
| Discount rate (annual) | 3% | Fixed | Thai HTA guidelines |
| Utility: Diagnosed OMG | 0.872 | ±20% | [17] |
| Utility: Undiagnosed MG | 0.739 | ±20% | [17] |
| WTP threshold (THB/QALY) | 160,000–200,000 | Fixed | [15,16] |

This table summarizes the input assumptions for epidemiologic, diagnostic, cost, and utility variables used in the decision-analytic model. Parameter ranges reflect ±20% variation from base-case estimates unless otherwise specified.

For probabilistic sensitivity analysis (PSA), beta distributions were assigned to probabilities and utilities, and gamma distributions to cost parameters.

**Abbreviations:** ACE, acquired comitant esotropia; AChR-Ab, acetylcholine receptor antibody; HTA, health technology assessment; MG, myasthenia gravis; OMG, ocular myasthenia gravis; QALY, quality-adjusted life year; TFT, thyroid function test; TSH, thyroid-stimulating hormone; FT3, free triiodothyronine; FT4, free thyroxine; THB, Thai Baht; WTP, willingness-to-pay.
