# Supplementary material for: A preliminary cost-utility analysis of routine myasthenia gravis and thyroid dysfunction screening in acquired comitant Esotropia
Source: PLoS One. 2026 May 28;21(5):e0350280. doi: 10.1371/journal.pone.0350280 (PMC13218454; doi:10.1371/journal.pone.0350280)
Supplement: S4a Table — Probabilities and utilities were modeled using beta distributions, parameterized by α = p × n and β = (1 – p) × n. For instance, a sensitivity of 75% (p = 0.75) with an assumed sample size of n = 100 yields α = 75, β = 25. Cost parameters followed gamma distributions, defined by shape (k = μ²/σ²) and scale (θ = σ²/μ), assuming variance ±20% of the mean in the absence of empirical data. This method adheres to standard practice for health economic modeling and PSA in line with Thai HTA recommendations. (DOCX) [file pone.0350280.s008.docx]

**S4a Table. Distribution Parameters for Probabilistic Sensitivity Analysis**

| Parameter | Distribution Type | Parameters | Notes |
| --- | --- | --- | --- |
| AChR-Ab sensitivity | Beta | α = 75, β = 25 | Derived from 75% sensitivity assumption |
| AChR-Ab specificity | Beta | α = 98, β = 2 | Derived from 98% specificity assumption |
| TFT sensitivity | Beta | α = 90, β = 10 | From diagnostic accuracy studies |
| TFT specificity | Beta | α = 92, β = 8 | From diagnostic accuracy studies |
| Utility: Diagnosed OMG | Beta | α = 87, β = 13 | Centered at 0.872 with variance ±20% |
| Utility: Undiagnosed MG | Beta | α = 74, β = 26 | Centered at 0.739 with variance ±20% |
| Cost variables | Gamma | k = mean²/var, θ = var/mean | Parameters calculated from hospital cost data |

Probabilities and utilities were modeled using beta distributions, parameterized by α = p × n and β = (1 – p) × n. For instance, a sensitivity of 75% (p=0.75) with an assumed sample size of n=100 yields α=75, β=25.

Cost parameters followed gamma distributions, defined by shape (k = μ²/σ²) and scale (θ = σ²/μ), assuming variance ±20% of the mean in the absence of empirical data. This method adheres to standard practice for health economic modeling and PSA in line with Thai HTA recommendations.
