# Supplementary material for: A preliminary cost-utility analysis of routine myasthenia gravis and thyroid dysfunction screening in acquired comitant Esotropia
Source: PLoS One. 2026 May 28;21(5):e0350280. doi: 10.1371/journal.pone.0350280 (PMC13218454; doi:10.1371/journal.pone.0350280)
Supplement: S1 File — Completed Consolidated Health Economic Evaluation Reporting Standards (CHEERS 2022) checklist for this study, indicating where each reporting item is addressed in the manuscript. (DOCX) [file pone.0350280.s009.docx]

# CHEERS 2022 Checklist – Completed for This Manuscript

| Item | Recommendation | Reported in section |
| --- | --- | --- |
| Title | Identify the study as an economic evaluation and specify the interventions being compared. | Title – identifies as cost–utility analysis of AChR-Ab and TFT screening vs. no screening (symptom-triggered testing) |
| Abstract | Provide a structured summary with context, key methods, results and sensitivity analyses. | Abstract – structured with purpose, methods, results, conclusion |
| Background and objectives | Give the context, study question and relevance for policy/practice. | Introduction – ACE, MG, TED context, rationale for screening, policy relevance |
| Health economic analysis plan | Indicate whether a formal HE analysis plan was developed. | Methods – retrospective design, decision-analytic modeling; no standalone protocol |
| Study population | Describe characteristics of the study population. | Methods – 110 ACE patients, demographics and clinical details |
| Setting and location | Provide relevant contextual information. | Methods – tertiary Thai hospital, HTA guideline context |
| Comparators | Describe the interventions or strategies being compared and why chosen. | Methods – no screening (symptom-triggered testing) vs. universal screening (AChR-Ab + TFT) |
| Perspective | State the perspective(s) adopted and why. | Methods – Thai healthcare system perspective, direct medical costs |
| Time horizon | State and justify time horizon. | Methods – 10 years to capture long-term outcomes |
| Discount rate | Report the discount rate and reason chosen. | Methods – 3% annually, per Thai HTA |
| Selection of outcomes | Describe outcome measures of benefits and harms. | Methods – QALYs, disutility of undiagnosed/delayed cases |
| Measurement of outcomes | Describe how outcomes were measured. | Methods – utilities from literature for MG, OMG, hypothyroidism, TED |
| Valuation of outcomes | Describe population and methods for utility valuation. | Methods – published international sources; Thai data unavailable |
| Measurement and valuation of resources and costs | Describe how costs were valued. | Methods – hospital billing, national cost references, CPI adjusted |
| Currency, price date, and conversion | Report unit cost year and currency. | Methods – Thai Baht, updated to 2024 values |
| Rationale and description of model | Describe model used and why; availability. | Methods – decision tree with 10-year Markov model, rationale explained |
| Analytics and assumptions | Describe analytic methods, assumptions, validation. | Methods – base-case, scenario analyses, PSA 10,000 iterations |
| Characterizing heterogeneity | Describe subgroup analyses. | Not performed; acknowledged in Discussion |
| Characterizing distributional effects | Describe distribution across individuals or priority populations. | Not applied; discussed in context of tertiary care |
| Characterizing uncertainty | Describe how uncertainty was addressed. | Methods – PSA, one-way sensitivity, tornado diagram |
| Approach to engagement | Describe engagement with patients/public/stakeholders. | Not applicable; retrospective data only |
| Study parameters | Report all analytic inputs and uncertainty assumptions. | Results – tables of prevalence, utilities, costs, sensitivity inputs |
| Summary of main results | Report mean values for main costs and outcomes. | Results – Table 3 (QALYs, costs, ICERs) |
| Effect of uncertainty | Report how uncertainty affects findings. | Results – PSA Figures 2–3, one-way sensitivity Figure 4 |
| Effect of engagement | Report on impact of engagement on study design/findings. | Not applicable |
| Study findings, limitations, generalizability, current knowledge | Report findings, limitations, ethical or equity issues, impact. | Discussion – key results, limitations (small sample, non-Thai utilities), policy implications |
| Source of funding | Describe study funding and funder role. | Unfunded |
| Conflicts of interest | Report conflicts of interest. | Authors declare none |

Husereau D, Drummond M, Augustovski F, de Bekker-Grob E, Briggs AH, Carswell C, Caulley L, Chaiyakunapruk N, Greenberg D, Loder E, Mauskopf J, Mullins CD, Petrou S, Pwu RF, Staniszewska S; CHEERS 2022 ISPOR Good Research Practices Task Force. Consolidated Health Economic Evaluation Reporting Standards 2022 (CHEERS 2022) Statement: Updated Reporting Guidance for Health Economic Evaluations. BMJ. 2022;376:e067975. The checklist is Open Access distributed in accordance with the terms of the Creative Commons Attribution (CC BY 4.0) license, which permits others to distribute, remix, adapt and build upon this work, for commercial use, provided the original work is properly cited. See: http://creativecommons.org/licenses/by/4.0/.
