# Supplementary material for: A preliminary cost-utility analysis of routine myasthenia gravis and thyroid dysfunction screening in acquired comitant Esotropia
Source: PLoS One. 2026 May 28;21(5):e0350280. doi: 10.1371/journal.pone.0350280 (PMC13218454; doi:10.1371/journal.pone.0350280)
Supplement: Attachment — Submitted filename: Response to Reviewers.docx [file pone.0350280.s011.docx]

**Response Letter**

PONE-D-25-52845

A Preliminary Cost-Utility Analysis of Routine Myasthenia Gravis and Thyroid Dysfunction Screening in Acquired Comitant Esotropia: An Exploratory Study

PLOS One

Dear Dr. Srimanan,

Thank you for submitting your manuscript to PLOS ONE. After careful consideration, we feel that it has merit but does not fully meet PLOS ONE’s publication criteria as it currently stands. Therefore, we invite you to submit a revised version of the manuscript that addresses the points raised during the review process.

Please submit your revised manuscript by May 16 2026 11:59PM. If you will need more time than this to complete your revisions, please reply to this message or contact the journal office at [plosone@plos.org](mailto:plosone@plos.org). When you're ready to submit your revision, log on to <https://www.editorialmanager.com/pone/> and select the 'Submissions Needing Revision' folder to locate your manuscript file.

Please include the following items when submitting your revised manuscript:

- A letter that responds to each point raised by the academic editor and reviewer(s). You should upload this letter as a separate file labeled 'Response to Reviewers'.
- A marked-up copy of your manuscript that highlights changes made to the original version. You should upload this as a separate file labeled 'Revised Manuscript with Track Changes'.
- An unmarked version of your revised paper without tracked changes. You should upload this as a separate file labeled 'Manuscript'.

If you would like to make changes to your financial disclosure, please include your updated statement in your cover letter. Guidelines for resubmitting your figure files are available below the reviewer comments at the end of this letter.

If applicable, we recommend that you deposit your laboratory protocols in [protocols.io](http://protocols.io/) to enhance the reproducibility of your results. Protocols.io assigns your protocol its own identifier (DOI) so that it can be cited independently in the future. For instructions see: [https://journals.plos.org/plosone/s/submission-guidelines#loc-laboratory-protocols](https://track.editorialmanager.com/CL0/https:%2F%2Fjournals.plos.org%2Fplosone%2Fs%2Fsubmission-guidelines%23loc-laboratory-protocols/1/010f019d4d7f7179-7365acfc-96e8-44e9-92dc-2008a7728622-000000/MyJ5fQLPm-ic18ffOG4fnKeHRRMfw5fZF_SH5qEvraE=257). Additionally, PLOS ONE offers an option for publishing peer-reviewed Lab Protocol articles, which describe protocols hosted on [protocols.io](http://protocols.io/). Read more information on sharing protocols at [https://plos.org/protocols?utm_medium=editorial-email&utm_source=authorletters&utm_campaign=protocols](https://track.editorialmanager.com/CL0/https:%2F%2Fplos.org%2Fprotocols%3Futm_medium=editorial-email%26utm_source=authorletters%26utm_campaign=protocols/1/010f019d4d7f7179-7365acfc-96e8-44e9-92dc-2008a7728622-000000/Qh3c2xB3UkM3DoGmE3VcTHhl7Q0f1uoPgFpTp42z-QA=257).

As the corresponding author, your ORCID iD is verified in the submission system and will appear in the published article. PLOS supports the use of ORCID, and we encourage all coauthors to register for an ORCID iD and use it as well. Please encourage your coauthors to verify their ORCID iD within the submission system before final acceptance, as unverified ORCID iDs will not appear in the published article. *Only* the individual author can complete the verification step; PLOS staff *cannot* verify ORCID iDs on behalf of authors.

We look forward to receiving your revised manuscript.

Kind regards,

Alejandro Torrado Pacheco, PhD
Associate Editor
PLOS One

Journal Requirements:

When submitting your revision, we need you to address these additional requirements.

1.Please ensure that your manuscript meets PLOS ONE's style requirements, including those for file naming. The PLOS ONE style templates can be found at

<https://journals.plos.org/plosone/s/file?id=wjVg/PLOSOne_formatting_sample_main_body.pdf> and

[https://journals.plos.org/plosone/s/file?id=ba62/PLOSOne_formatting_sample_title_authors_affiliations.pdf](https://track.editorialmanager.com/CL0/https:%2F%2Fjournals.plos.org%2Fplosone%2Fs%2Ffile%3Fid=ba62%2FPLOSOne_formatting_sample_title_authors_affiliations.pdf/1/010f019d4d7f7179-7365acfc-96e8-44e9-92dc-2008a7728622-000000/0ZofqawGScXlBP3HlVXN52FL6ck0d-OxpBJ5ZHtEo1I=257)

**Response:** Thank you for this guidance.

We have revised the manuscript to ensure full compliance with PLOS ONE formatting and style requirements. The manuscript has been aligned with the journal’s templates for structure, title page, author affiliations, figure and table presentation, and reference formatting. File naming conventions have also been updated accordingly.

We have systematically reviewed the submission against the provided PLOS ONE templates to ensure accuracy, consistency, and completeness.

2. Please note that PLOS One has specific guidelines on code sharing for submissions in which author-generated code underpins the findings in the manuscript. In these cases, all author-generated code must be made available without restrictions upon publication of the work. Please review our guidelines at <https://journals.plos.org/plosone/s/materials-and-software-sharing#loc-sharing-code> and ensure that your code is shared in a way that follows best practice and facilitates reproducibility and reuse.

**Response:** Thank you for highlighting the journal’s code-sharing policy.

This study did not involve author-generated code. The decision tree and Markov model were constructed using standard spreadsheet-based modeling (Microsoft Excel) without the use of custom programming scripts.

To ensure reproducibility, all model structures, assumptions, input parameters, and cost components have been explicitly described in the Methods and Supplementary Materials. These details allow independent replication of the analysis without reliance on proprietary code.

Therefore, no additional code is available for sharing.

3. Your ethics statement should only appear in the Methods section of your manuscript. If your ethics statement is written in any section besides the Methods, please move it to the Methods section and delete it from any other section. Please ensure that your ethics statement is included in your manuscript, as the ethics statement entered into the online submission form will not be published alongside your manuscript.

**Response:** Thank you for this important clarification.

We have revised the manuscript to comply fully with PLOS ONE requirements. The ethics statement has been consolidated and placed exclusively within the Methods section (Page 7, lines 206–211). Any duplicate ethics-related content previously included in other sections (e.g., Declarations) has been removed.

The ethics statement now clearly includes institutional review board approval details and consent procedures, ensuring that it is complete and will be appropriately displayed in the published version of the manuscript.

4. Please upload a copy of Figure 6 to which you refer in your text on page 11. If the figure is no longer to be included as part of the submission please remove all reference to it within the text.

**Response:** Thank you for bringing this to our attention.

Figure 6 was not intended to be part of the final manuscript. We have removed all references to Figure 6 from the text and conducted a comprehensive review of all figure citations to ensure consistency and correct numbering throughout the manuscript.

We appreciate the editor’s careful review, which has helped improve the overall clarity and accuracy of the submission.

5. We note that there is identifying data in the Supporting Information file  Supplementary table 1.docx and Excel file AACE 2025.xlsx. Due to the inclusion of these potentially identifying data, we have removed this file from your file inventory. Prior to sharing human research participant data, authors should consult with an ethics committee to ensure data are shared in accordance with participant consent and all applicable local laws.

Data sharing should never compromise participant privacy. It is therefore not appropriate to publicly share personally identifiable data on human research participants. The following are examples of data that should not be shared:

-Name, initials, physical address

-Ages more specific than whole numbers

-Internet protocol (IP) address

-Specific dates (birth dates, death dates, examination dates, etc.)

-Contact information such as phone number or email address

-Location data

-ID numbers that seem specific (long numbers, include initials, titled “Hospital ID”) rather than random (small numbers in numerical order)

Data that are not directly identifying may also be inappropriate to share, as in combination they can become identifying. For example, data collected from a small group of participants, vulnerable populations, or private groups should not be shared if they involve indirect identifiers (such as sex, ethnicity, location, etc.) that may risk the identification of study participants.

Additional guidance on preparing raw data for publication can be found in our Data Policy (<https://journals.plos.org/plosone/s/data-availability#loc-human-research-participant-data-and-other-sensitive-data>) and in the following article: <http://www.bmj.com/content/340/bmj.c181.long>.

Please remove or anonymize all personal information (<specific identifying information in file to be removed>), ensure that the data shared are in accordance with participant consent, and re-upload a fully anonymized data set. Please note that spreadsheet columns with personal information must be removed and not hidden as all hidden columns will appear in the published file.

**Response:** Thank you for this important guidance regarding data privacy and sharing.

In accordance with PLOS ONE data policy, we have carefully revised all Supporting Information files to ensure full de-identification of participant data. All direct identifiers (e.g., hospital numbers, dates, and free-text information) have been permanently removed.

To further minimize the risk of indirect identification, we have reduced data granularity where appropriate. Specifically, age has been categorized into groups rather than reported as exact values, and variables with potential re-identification risk have been simplified or aggregated. In small subgroups (e.g., MG cases), we ensured that combinations of variables do not allow individual patients to be identifiable.

We confirm that all identifying columns have been permanently deleted (not hidden), and the revised dataset has been re-uploaded in a fully anonymized form. Data sharing is now compliant with participant confidentiality, institutional review board requirements, and applicable data protection standards.

We appreciate the editor’s guidance in improving the ethical integrity and transparency of our data presentation.

6. If the reviewer comments include a recommendation to cite specific previously published works, please review and evaluate these publications to determine whether they are relevant and should be cited. There is no requirement to cite these works unless the editor has indicated otherwise. 
**Response:** Thank you for this guidance.

We have carefully reviewed all references suggested during the peer review process and evaluated their relevance to the objectives and scope of our study. Relevant and appropriate citations have been incorporated into the revised manuscript where they strengthen the scientific context and support the interpretation of our findings.

We confirm that all references included in the revised manuscript have been selected based on their relevance and contribution to the topic, in accordance with journal recommendations.

Additional Editor Comments :

Please note that we have only been able to secure a single reviewer to assess your manuscript. We are issuing a decision on your manuscript at this point to prevent further delays in the evaluation of your manuscript. Please be aware that the editor who handles your revised manuscript might find it necessary to invite additional reviewers to assess this work once the revised manuscript is submitted. However, we will aim to proceed on the basis of this single review if possible. 

The reviewer requests several revisions, their comments are available below. Could you please address each comment carefully?

[Note: HTML markup is below. Please do not edit.]

Reviewers' comments:

Reviewer's Responses to Questions

**Response:** We sincerely thank the Editor and Reviewer for their careful and constructive evaluation of our manuscript. We appreciate the positive comments regarding the clinical relevance of the topic and the appropriateness of the decision-analytic approach. In response, we have revised the manuscript extensively to improve internal consistency, transparency of model assumptions, reproducibility of cost inputs, and caution in interpreting the results. We have also revised the abstract, main text, tables, figure legends, and supporting information accordingly.

All revisions have been carefully implemented in the revised manuscript with tracked changes. Page and line numbers below refer to the revised manuscript. These revisions collectively address all seven concerns raised by the reviewer, including clarification of comparator definition, consistency of economic results, transparency of model structure and costs, interpretation of QALYs, definition of targeted screening, acknowledgment of data limitations, and appropriate framing of conclusions.

**Comments to the Author**

1. Is the manuscript technically sound, and do the data support the conclusions?

The manuscript must describe a technically sound piece of scientific research with data that supports the conclusions. Experiments must have been conducted rigorously, with appropriate controls, replication, and sample sizes. The conclusions must be drawn appropriately based on the data presented.

Reviewer #1: Partly

2. Has the statistical analysis been performed appropriately and rigorously?

Reviewer #1: Yes

3. Have the authors made all data underlying the findings in their manuscript fully available?

The [PLOS Data policy](http://track.editorialmanager.com/CL0/http:%2F%2Fwww.plosone.org%2Fstatic%2Fpolicies.action%23sharing/1/010f019d4d7f7179-7365acfc-96e8-44e9-92dc-2008a7728622-000000/d6e1cq7I4TzZv5KzjvWxIUp5sF2mt0zOb66INI-FUtU=257) requires authors to make all data underlying the findings described in their manuscript fully available without restriction, with rare exception (please refer to the Data Availability Statement in the manuscript PDF file). The data should be provided as part of the manuscript or its supporting information, or deposited to a public repository. For example, in addition to summary statistics, the data points behind means, medians and variance measures should be available. If there are restrictions on publicly sharing data—e.g. participant privacy or use of data from a third party—those must be specified.

Reviewer #1: Yes

4. Is the manuscript presented in an intelligible fashion and written in standard English?

PLOS ONE does not copyedit accepted manuscripts, so the language in submitted articles must be clear, correct, and unambiguous. Any typographical or grammatical errors should be corrected at revision, so please note any specific errors here.

Reviewer #1: Yes

5. Review Comments to the Author

Please use the space provided to explain your answers to the questions above. You may also include additional comments for the author, including concerns about dual publication, research ethics, or publication ethics. (Please upload your review as an attachment if it exceeds 20,000 characters)

Reviewer #1: The manuscript addresses an important and clinically relevant question regarding the cost-effectiveness of screening for myasthenia gravis among patients presenting with ocular symptoms. The topic is timely, and the use of decision-analytic modeling with both deterministic and probabilistic sensitivity analyses is appropriate. This work is commendable! However, several areas require clarification and strengthening before the results can be interpreted with full confidence. I therefore recommend revision, primarily to improve transparency, internal consistency, and reproducibility.

1. Inconsistency in comparator strategy description:
There appears to be inconsistency in how the comparator strategy is described. The introduction refers to “universal screening versus symptom-triggered testing,” whereas other sections and the decision tree describe “no routine screening versus universal screening.” These sound like conceptually distinct strategies. Clarification is needed to ensure that the comparator pathway is consistently defined throughout the manuscript.

**Response:** We thank the reviewer for identifying this important inconsistency.

We agree that the comparator strategy must be clearly and uniformly defined throughout the manuscript. In our model, the intended comparator is a single strategy: no routine baseline screening with symptom-triggered testing during follow-up. Under this approach, patients do not undergo initial AChR-Ab or thyroid function testing at presentation but may receive diagnostic evaluation later if clinical suspicion arises.

We acknowledge that the previous wording (“symptom-triggered testing” vs. “no routine screening”) may have suggested conceptually distinct strategies. We have therefore clarified that these terms refer to the same comparator pathway and have standardized the terminology throughout the manuscript to: “no routine screening (symptom-triggered testing)”.

This terminology has been applied consistently in the Introduction, Methods (Diagnostic Strategies), Results, and the decision tree description to ensure conceptual alignment between the narrative and the modeled pathways.

These revisions improve clarity and ensure that the comparator strategy is consistently defined across all sections of the manuscript.

- (Page 1, lines 15–17) - Revised
- (Pages 2-3, lines 65–67) - Revised
- (Page 3, lines 89) - Rewritten (Diagnostic Strategies section)
- (Page 8, lines 255) - Standardized wording in Results

2. Inconsistency between the base case, one-way sensitivity analysis, and probabilistic sensitivity analysis (PSA):
There appears to be some inconsistency between the base-case results and the PSA. In the base case, universal screening is reported to generate higher costs and higher QALYs, suggesting that screening is more effective but more costly than no screening. However, the PSA section notes that most simulations fall in the northwestern quadrant (more costly and less effective). If screening is generally less effective and more costly in the PSA, this would not align with the base-case conclusion. Clarifying this apparent discrepancy would help readers better understand how the PSA results relate to the base-case findings.

Similarly, there is a difference between the base-case ICER and the one-way sensitivity analysis results. The base-case ICER (285,360 baht per QALY) is above Thailand’s willingness-to-pay threshold of 160,000–200,000 baht per QALY, which suggests that screening is not cost-effective in the base case. However, the manuscript states that the net monetary benefit (NMB) remained positive at a WTP of 160,000 baht per QALY. Since NMB and ICER are mathematically linked, additional clarification on the WTP value used for the NMB calculation or on the interpretation of these results would be helpful to ensure internal consistency.

**Response:** We thank the reviewer for this important observation and agree that internal consistency between the base-case, one-way sensitivity analysis, and probabilistic sensitivity analysis (PSA) is essential.

In the revised manuscript, we have clarified the base-case analysis as the primary reference, incorporating real-world diagnostic accuracy assumptions. Under this base case, universal screening resulted in higher costs and higher QALYs compared with no routine screening (symptom-triggered testing), with an ICER of ฿285,360 per QALY, exceeding the Thai willingness-to-pay (WTP) threshold of ฿160,000–200,000/QALY. The interpretation has been revised accordingly to state that universal screening is not cost-effective under current cost structures.

We identified that the previous description of the PSA contained an error. The statement referring to the “northwestern quadrant” has been corrected to “northeastern quadrant,” consistent with the simulation results and reflecting that screening is generally more effective but more costly. The PSA results and corresponding figure legend have been revised to ensure alignment with the base-case findings.

We also addressed the inconsistency between ICER and net monetary benefit (NMB). Given that the base-case ICER exceeds the WTP threshold, the corresponding NMB at ฿160,000/QALY is negative. The previous statement indicating positive NMB has therefore been removed. The one-way sensitivity analysis section has been revised to report parameter influence without implying cost-effectiveness.

These revisions ensure that the base-case analysis, sensitivity analyses, and PSA results are now conceptually and mathematically consistent throughout the manuscript.

- (Page 1, lines 26–28) - Revised PSA quadrant (NW → NE)
- (Page 12, lines 355–358) - Rewritten PSA interpretation
- (Page 6, lines 190–195) - Revised NMB description (no positive claim)
- (Page 12, lines 355–358) - Aligned PSA with base-case

3. Decision tree:
The decision tree indicates delayed diagnosis downstream, and additional follow-up or confirmatory evaluations are modeled downstream. However, the manuscript does not clearly report the costs associated with these additional visits, diagnostic tests, or confirmatory procedures. If these elements are incorporated structurally in the model, their associated costs should be explicitly presented in the cost inputs table. MG and hypothyroidism treatment costs have also not been mentioned. Greater transparency is needed to ensure the analysis is reproducible.

**Response:** We thank the reviewer for this important comment regarding the transparency of downstream costs.

We agree that the original manuscript did not sufficiently detail the cost components associated with follow-up, confirmatory evaluation, and treatment within the decision tree structure. In the revised manuscript, we have clarified that all downstream pathways—including true-positive, false-negative, false-positive, and comparator (no routine screening) cases—incorporate associated medical costs.

Specifically, we have explicitly described in the Methods (Cost Inputs section) that the model includes costs for outpatient follow-up visits, confirmatory investigations (e.g., chest CT for thymoma evaluation and additional diagnostic work-up where clinically indicated), and disease-specific treatments (e.g., pyridostigmine for myasthenia gravis and levothyroxine for hypothyroidism). These costs are now clearly itemized and quantified.

In addition, we have added and cross-referenced a detailed supplementary cost table (S3 Table), which provides a transparent breakdown of all cost components, including unit costs, quantities, and their application within the model.

We have also revised the description of the decision tree to clarify that each diagnostic outcome pathway (true positive, false negative, false positive, and true negative) is linked to corresponding downstream costs and health outcomes.

These revisions improve transparency, ensure consistency between the model structure and reported inputs, and enhance reproducibility of the analysis.

- (Page 5, lines 138–140) - Added downstream cost linkage in decision tree
- (Page 5, lines 147–151) - Added explicit cost components (follow-up, CT, treatment)
- (Page 5, lines 156–159) - Added reference to Supplementary Table 3

4. Magnitude of QALY gains in scenario analysis:
The scenario analysis reports about 933 QALYs gained for 110 patients over 10 years, or roughly 8.5 QALYs per patient, suggesting most patients remain in near-perfect health. For a population with myasthenia gravis, diplopia, strabismus burden, steroid-related adverse effects, risk of generalization, and potential crises, this estimate seems optimistic. The manuscript would benefit from a clear presentation of health-state utility values, a description of how utilities change over time, and a justification for the long-term quality of life assumption.

**Response:** We thank the reviewer for this thoughtful observation regarding the magnitude of QALY estimates in the scenario analysis.

We agree that the reported total QALYs may appear high if interpreted as reflecting disease-specific burden across all patients. However, we would like to clarify that these values represent cohort-level, population-average QALYs over a 10-year time horizon. In our cohort, only a small proportion of patients had myasthenia gravis (2.7%) or thyroid dysfunction (0.9%), while the majority of patients remained in health states close to full utility. As a result, the overall QALY estimates are driven primarily by individuals without systemic disease, leading to high average utility values over time.

To improve clarity, we have revised the manuscript to explicitly distinguish between absolute cohort-level QALYs and incremental QALY differences between strategies, emphasizing that the latter is the key measure for cost-effectiveness interpretation.

We have also expanded the Methods section to more clearly describe health-state utility values and their assignment across diagnostic pathways, including early diagnosis, delayed diagnosis, and treated states. These utility inputs are now more explicitly referenced to Supplementary Table 2.

Furthermore, we have clarified in the Results and Discussion that the scenario analysis reflects less conservative assumptions (i.e., unadjusted utilities without incorporating diagnostic uncertainty), and therefore represents an optimistic scenario rather than real-world estimates.

Finally, we have strengthened the limitations section to acknowledge that long-term utility assumptions may overestimate health-related quality of life, and that utility values were derived from published studies rather than Thai population data.

These revisions aim to improve transparency and ensure appropriate interpretation of QALY outcomes within the context of the modeled population.

- (Page 6, lines 185–187) - Added clarification on cohort-level QALYs
- (Page 9, lines 269–274) - Rewritten scenario interpretation
- (Page 14, lines 416–420) - Expanded discussion explanation

5. Targeted screening strategy:
The targeted screening strategy would benefit from a clearer operational definition. While examples of high-risk features are provided, the manuscript does not fully describe how the 30% high-risk subgroup is identified in the model, whether this subgroup has a higher disease prevalence than the overall population, or whether the accuracy of the targeting approach was incorporated. Providing more detail on these assumptions would improve reproducibility and transparency

**Response:** We thank the reviewer for this important comment and agree that the targeted screening strategy required a clearer operational definition to ensure transparency and reproducibility.

In the revised manuscript, we have clarified that the targeted screening analysis was designed as an exploratory scenario reflecting a pragmatic referral-center approach, rather than a formally validated clinical prediction rule. Specifically, we assumed that 30% of patients with acquired comitant esotropia (ACE) would be classified as “higher risk” based on the presence of atypical clinical features, including adult-onset diplopia, rapid symptom onset, or variability in ocular deviation.

We further clarify that this 30% proportion was an assumption informed by referral patterns reported in the literature, rather than being directly derived from our cohort data. Importantly, in the current model, we did not assign a different underlying disease prevalence to the targeted subgroup, nor did we model a separate targeting test with its own sensitivity or specificity. Instead, the targeted strategy was implemented as a proportional application of the universal screening pathway to a predefined subset of the cohort, with identical diagnostic performance and downstream cost–utility assumptions.

To improve clarity, we have revised the Methods section to explicitly describe these modeling assumptions, and we have updated the Results and Discussion sections to emphasize that the targeted screening analysis should be interpreted as exploratory rather than definitive. We have also expanded the limitations section to acknowledge that the absence of a validated risk-stratification rule and the assumption of uniform disease prevalence across subgroups may limit the generalizability of this approach.

We agree that future studies should aim to develop and validate a formal clinical prediction model with defined sensitivity and specificity, which could then be incorporated into a more rigorous cost-effectiveness framework.

- (Page 4, lines 107–112) - Added operational definition + assumptions
- (Page 4, lines 113–117) - Added clarification paragraph
- (Page 11, lines 319–322) - Revised interpretation (efficiency clarified)
- (Page 13, lines 409–415) - Added limitation (no differential prevalence / no model accuracy)

6. Preliminary analysis, but data insufficient to support policy conclusions:
The analysis is also based on a small number of observed events (3 myasthenia gravis cases and 1 hypothyroid case). With such low event counts, the ICER may be sensitive to small changes in prevalence (as mentioned in the study limitation). While the analysis may be considered preliminary, the current data are insufficient to support policy-definitive conclusions.

**Response:** We thank the reviewer for this important comment and fully agree that the small number of observed outcome events limits the strength of policy-level inferences.

In our cohort, only three cases of myasthenia gravis and one case of hypothyroidism were identified, resulting in low event rates. As a consequence, model outputs—particularly the incremental cost-effectiveness ratio (ICER)—are sensitive to small changes in prevalence and underlying assumptions. We acknowledge that this introduces uncertainty in the stability and generalizability of the results.

In the revised manuscript, we have emphasized more clearly that this study represents a preliminary and exploratory cost–utility analysis. We have revised the Abstract, Introduction, Discussion, and Conclusion to avoid policy-definitive language and to frame the findings as hypothesis-generating. Specifically, we now state that the results should not be interpreted as sufficient to guide immediate policy implementation, but rather as an initial quantitative framework to inform future research.

We have also strengthened the limitations section to explicitly acknowledge that the small number of outcome events may affect the precision of prevalence estimates and the robustness of economic conclusions. Accordingly, we emphasize the need for larger, prospective, and multicenter studies to validate these findings and to provide more stable inputs for cost-effectiveness modeling.

These revisions were made to ensure that the conclusions are appropriately aligned with the available data and do not overstate the policy implications of this preliminary analysis.

- (Page 2, lines 32–35) - Abstract softened (“preliminary”)
- (Page 3, lines 69–71) - Added exploratory framing in Introduction
- (Page 15, lines 486–490) - Expanded limitation (small event numbers)
- (Page 16, lines 509–512) - Conclusion softened (no policy claim)

7. Implementation cost consideration:
While test costs are included, it is unclear whether infrastructure, laboratory setup, and implementation costs were considered. Even if these costs are ultimately minimal, clarifying their inclusion or exclusion would improve transparency.

**Response:** We thank the reviewer for this important comment regarding implementation and infrastructure costs.

In the revised manuscript, we have clarified that the analysis was conducted from the Thai healthcare sector perspective, which includes direct medical costs related to patient care (e.g., diagnostic tests, follow-up visits, confirmatory investigations, and treatment). In accordance with this perspective, capital and implementation costs—such as laboratory platform acquisition, assay setup, quality-control programs, and personnel training—were not explicitly included in the model.

We have now explicitly stated this in both the Methods and Discussion sections to improve transparency. We also acknowledge that excluding these costs may underestimate the true economic impact of implementing universal screening, particularly in settings where laboratory infrastructure is not already established.

However, in tertiary-care centers such as our institution, where AChR-Ab and thyroid function testing are already routinely available, these infrastructure costs are largely sunk or shared across multiple clinical services. Therefore, their exclusion is unlikely to materially alter the relative cost-effectiveness comparison between strategies in this context.

We have incorporated this clarification into the limitations section and emphasized that the generalizability of our findings to lower-resource settings may be limited. Future studies adopting a broader health system or societal perspective should explicitly incorporate implementation and infrastructure costs to better inform policy-level decision-making.

These revisions were made to ensure consistency between the analytic perspective and the included cost components, and to enhance transparency and interpretability of the economic analysis.

- (Pages 8-9, lines 260–264) - Clarified healthcare-sector perspective + exclusion of implementation costs
- (Page 15, lines 460–467) - Discussion: impact of infrastructure costs explained
- (Pages 15-16, lines 492–497) - Limitations: explicit statement of exclusion

6. PLOS authors have the option to publish the peer review history of their article ([what does this mean?](https://track.editorialmanager.com/CL0/https:%2F%2Fjournals.plos.org%2Fplosone%2Fs%2Feditorial-and-peer-review-process%23loc-peer-review-history/1/010f019d4d7f7179-7365acfc-96e8-44e9-92dc-2008a7728622-000000/an_Co-3d9A57dS-zMuC3qbmUwmsyCkAVjbTCQ86A6D8=257)). If published, this will include your full peer review and any attached files.


If you choose “no”, your identity will remain anonymous but your review may still be made public.


**Do you want your identity to be public for this peer review?** For information about this choice, including consent withdrawal, please see our [Privacy Policy](https://track.editorialmanager.com/CL0/https:%2F%2Fwww.plos.org%2Fprivacy-policy/1/010f019d4d7f7179-7365acfc-96e8-44e9-92dc-2008a7728622-000000/snSUiGlKuCjSLOTYnjuRDsU5F6ib6aUzHaAML2PB1H4=257).

Reviewer #1: **Yes:** Rashmi Paudel

[NOTE: If reviewer comments were submitted as an attachment file, they will be attached to this email and accessible via the submission site. Please log into your account, locate the manuscript record, and check for the action link "View Attachments". If this link does not appear, there are no attachment files.]

To ensure your figures meet our technical requirements, please review our figure guidelines: <https://journals.plos.org/plosone/s/figures>

You may also use PLOS’s free figure tool, NAAS, to help you prepare publication quality figures: <https://journals.plos.org/plosone/s/figures#loc-tools-for-figure-preparation>.

NAAS will assess whether your figures meet our technical requirements by comparing each figure against our figure specifications.
