# Supplementary material for: A preliminary cost-utility analysis of routine myasthenia gravis and thyroid dysfunction screening in acquired comitant Esotropia
Source: PLoS One. 2026 May 28;21(5):e0350280. doi: 10.1371/journal.pone.0350280 (PMC13218454; doi:10.1371/journal.pone.0350280)
Supplement: Attachment — Submitted filename: Response_to_reviewers_auresp_2.docx [file pone.0350280.s012.docx]

**Response to reviewers PLOS ONE second revision**

PONE-D-25-52845R1

A Preliminary Cost-Utility Analysis of Routine Myasthenia Gravis and Thyroid Dysfunction Screening in Acquired Comitant Esotropia

PLOS One

Dear Dr. Srimanan,

Thank you for submitting your manuscript to PLOS ONE. After careful consideration, we feel that it has merit but does not fully meet PLOS ONE’s publication criteria as it currently stands. Therefore, we invite you to submit a revised version of the manuscript that addresses the points raised during the review process.

Please submit your revised manuscript by Jun 10 2026 11:59PM. If you will need more time than this to complete your revisions, please reply to this message or contact the journal office at [plosone@plos.org](mailto:plosone@plos.org). When you're ready to submit your revision, log on to <https://www.editorialmanager.com/pone/> and select the 'Submissions Needing Revision' folder to locate your manuscript file.

Please include the following items when submitting your revised manuscript:

- A letter that responds to each point raised by the academic editor and reviewer(s). You should upload this letter as a separate file labeled 'Response to Reviewers'.
- A marked-up copy of your manuscript that highlights changes made to the original version. You should upload this as a separate file labeled 'Revised Manuscript with Track Changes'.
- An unmarked version of your revised paper without tracked changes. You should upload this as a separate file labeled 'Manuscript'.

If you would like to make changes to your financial disclosure, please include your updated statement in your cover letter. Guidelines for resubmitting your figure files are available below the reviewer comments at the end of this letter.

If applicable, we recommend that you deposit your laboratory protocols in [protocols.io](http://protocols.io/) to enhance the reproducibility of your results. Protocols.io assigns your protocol its own identifier (DOI) so that it can be cited independently in the future. For instructions see: [https://journals.plos.org/plosone/s/submission-guidelines#loc-laboratory-protocols](https://track.editorialmanager.com/CL0/https:%2F%2Fjournals.plos.org%2Fplosone%2Fs%2Fsubmission-guidelines%23loc-laboratory-protocols/1/010f019dcd0cc8ba-300440b9-49f3-4912-8ed4-f7e00177051b-000000/9fwn0zeJB59Ea39nb5-TNOhIi4uG7gr3Szm_a-YQq5M=258). Additionally, PLOS ONE offers an option for publishing peer-reviewed Lab Protocol articles, which describe protocols hosted on [protocols.io](http://protocols.io/). Read more information on sharing protocols at [https://plos.org/protocols?utm_medium=editorial-email&utm_source=authorletters&utm_campaign=protocols](https://track.editorialmanager.com/CL0/https:%2F%2Fplos.org%2Fprotocols%3Futm_medium=editorial-email%26utm_source=authorletters%26utm_campaign=protocols/1/010f019dcd0cc8ba-300440b9-49f3-4912-8ed4-f7e00177051b-000000/8GZkEM2YnLZhp_gDFh43kuwinhWn1-DR4bwe6yFAVFs=258).

As the corresponding author, your ORCID iD is verified in the submission system and will appear in the published article. PLOS supports the use of ORCID, and we encourage all coauthors to register for an ORCID iD and use it as well. Please encourage your coauthors to verify their ORCID iD within the submission system before final acceptance, as unverified ORCID iDs will not appear in the published article. *Only* the individual author can complete the verification step; PLOS staff *cannot* verify ORCID iDs on behalf of authors.

We look forward to receiving your revised manuscript.

Kind regards,

Redoy Ranjan, MS (CV&TS), Ch.M. (Edin), PhD
Academic Editor
PLOS One

Journal Requirements:

1. If the reviewer comments include a recommendation to cite specific previously published works, please review and evaluate these publications to determine whether they are relevant and should be cited. There is no requirement to cite these works unless the editor has indicated otherwise. 

2. Please review your reference list to ensure that it is complete and correct. If you have cited papers that have been retracted, please include the rationale for doing so in the manuscript text, or remove these references and replace them with relevant current references. Any changes to the reference list should be mentioned in the rebuttal letter that accompanies your revised manuscript. If you need to cite a retracted article, indicate the article’s retracted status in the References list and also include a citation and full reference for the retraction notice.

[Note: HTML markup is below. Please do not edit.]

Reviewers' comments:

Reviewer's Responses to Questions

**Comments to the Author**

1. If the authors have adequately addressed your comments raised in a previous round of review and you feel that this manuscript is now acceptable for publication, you may indicate that here to bypass the “Comments to the Author” section, enter your conflict of interest statement in the “Confidential to Editor” section, and submit your "Accept" recommendation.

Reviewer #1: All comments have been addressed

Reviewer #2: (No Response)

Reviewer #3: (No Response)

2. Is the manuscript technically sound, and do the data support the conclusions?

The manuscript must describe a technically sound piece of scientific research with data that supports the conclusions. Experiments must have been conducted rigorously, with appropriate controls, replication, and sample sizes. The conclusions must be drawn appropriately based on the data presented.

Reviewer #1: Yes

Reviewer #2: Partly

Reviewer #3: (No Response)

3. Has the statistical analysis been performed appropriately and rigorously?

Reviewer #1: Yes

Reviewer #2: Yes

Reviewer #3: (No Response)

4. Have the authors made all data underlying the findings in their manuscript fully available?

The [PLOS Data policy](http://track.editorialmanager.com/CL0/http:%2F%2Fwww.plosone.org%2Fstatic%2Fpolicies.action%23sharing/1/010f019dcd0cc8ba-300440b9-49f3-4912-8ed4-f7e00177051b-000000/HQijLwVP32S77U7-cKdvyyaHNCxIAtPf6B6KN-6H-V4=258) requires authors to make all data underlying the findings described in their manuscript fully available without restriction, with rare exception (please refer to the Data Availability Statement in the manuscript PDF file). The data should be provided as part of the manuscript or its supporting information, or deposited to a public repository. For example, in addition to summary statistics, the data points behind means, medians and variance measures should be available. If there are restrictions on publicly sharing data—e.g. participant privacy or use of data from a third party—those must be specified.

Reviewer #1: Yes

Reviewer #2: Yes

Reviewer #3: (No Response)

5. Is the manuscript presented in an intelligible fashion and written in standard English?

PLOS ONE does not copyedit accepted manuscripts, so the language in submitted articles must be clear, correct, and unambiguous. Any typographical or grammatical errors should be corrected at revision, so please note any specific errors here.

Reviewer #1: Yes

Reviewer #2: Yes

Reviewer #3: (No Response)

6. Review Comments to the Author

Please use the space provided to explain your answers to the questions above. You may also include additional comments for the author, including concerns about dual publication, research ethics, or publication ethics. (Please upload your review as an attachment if it exceeds 20,000 characters)

Reviewer #1: Thank you for the thorough revision of the manuscript. The authors have carefully and comprehensively addressed the concerns raised in the previous round of review. In particular, the revisions have improved the clarity and consistency of the comparator strategy, resolved internal inconsistencies between the base-case and sensitivity analyses, and enhanced transparency regarding model structure, cost inputs, and underlying assumptions.

The manuscript is now technically sound, with appropriate statistical and modeling approaches. The interpretation of findings has been appropriately tempered to reflect the preliminary nature of the analysis, especially given the small number of observed outcome events. The clarification of cohort-level QALY estimates and the explicit framing of the targeted screening strategy as exploratory are particularly helpful and improve the overall rigor and transparency of the study.

The conclusions are supported by the data presented and are now appropriately cautious, avoiding overinterpretation or policy-definitive claims. The manuscript is clearly written and presented in a logical and intelligible manner.

Overall, I believe the manuscript is suitable for publication in its current form.

Response: We sincerely thank the reviewer for the thoughtful and encouraging comments on our revised manuscript. We greatly appreciate the reviewer’s time and effort in evaluating our work.

We are grateful for your recognition that the revisions have improved the clarity, consistency, and transparency of the manuscript, particularly with respect to the comparator strategy, economic analyses, and model assumptions. We are also pleased that the interpretation of findings and the framing of the study as preliminary and exploratory are now considered appropriate.

Your positive feedback regarding the rigor, clarity, and overall presentation of the manuscript is highly appreciated. We are encouraged that the conclusions are now aligned with the data and appropriately cautious.

We thank you again for your constructive guidance throughout the review process, which has substantially strengthened the quality of our work.

Reviewer #2: The revision addresses most of the prior concerns: the comparator is now defined consistently, the PSA quadrant error was corrected, downstream costs are better described, the targeted-screening assumptions are stated more plainly, and the conclusions are appropriately softened as preliminary. Those are meaningful improvements.

However, a few residual problems remain. The targeted-screening arm is still largely a cost-reduction exercise rather than a true risk-enriched strategy, so its apparent efficiency can be overread. The scenario analysis still reports very high absolute QALYs and remains of limited interpretive value. There is also lingering internal inconsistency in diagnostic performance reporting, with AChR-Ab specificity given as 99% in the main text but 0.98 in the supplementary figure legend/discussion. In addition, wording around consent/publication and some prose remain awkward and should be cleaned before publication.

COMMENT 1 — Targeted screening still weak

We thank the reviewer for this important clarification. We agree that the targeted screening strategy in the current model does not represent a true risk-enriched approach, as no differential disease prevalence or targeting performance (e.g., sensitivity/specificity) was modeled.

To address this, we have further clarified in the Discussion that the observed improvement in cost-effectiveness reflects a reduction in testing volume rather than enrichment of disease probability. We have also explicitly emphasized that this strategy should not be interpreted as a validated or implementable screening approach, but rather as an exploratory scenario intended to illustrate the potential economic impact of selective testing.

These clarifications have been added to improve interpretability and to avoid overreading of the targeted screening results.

We added more discussion to the revised discussion section on page 13, lines 414-416.

COMMENT 2 — Scenario analysis QALYs too high

Response: We appreciate this insightful comment. We agree that the absolute QALY values reported in the scenario analysis are high and may be of limited interpretive value if considered in isolation.

In the revised manuscript, we have further clarified that these values represent cohort-level population averages over a 10-year horizon, in which the majority of patients remain in high-utility health states. We have also emphasized that the scenario analysis is based on optimistic assumptions and should not be interpreted as reflecting real-world disease burden.

To improve clarity, we have strengthened the wording in both the Results and Discussion sections to focus on incremental differences rather than absolute QALY values.

We rewrite the manuscript. Replace:

“rather than representing disease-specific outcomes…”

With:

“and therefore have limited interpretive value beyond illustrating relative differences between strategies under optimistic assumptions.” on page 9, lines 272-273.

COMMENT 3 — AChR-Ab specificity inconsistency (99% vs 0.98)

Response: We thank the reviewer for identifying this inconsistency. We have carefully reviewed the manuscript and supplementary materials and standardized the specificity of AChR-Ab testing to 0.98 (98%) across all sections, including the main text, tables, and figure legends.

These revisions ensure internal consistency in the reporting of diagnostic performance parameters.

We change the term to “specificity of approximately 98–99%” on page 2, line 53 of the introduction section of the manuscript. We replace all values with 0.98 in the materials and methods section on page 4, line 121. We also update page 13, line 401; page 14, line 424; and page 14, line 442 in the discussion section of the manuscript.

COMMENT 4 — Consent/wording awkward

Response: We thank the reviewer for this comment. We have carefully revised the manuscript to improve clarity and readability, including wording related to ethical approval, consent, and the overall prose throughout.

We Replace:

“Written informed consent for publication was waived by The Institutional Review Board…”

with:

“The requirement for written informed consent was waived by the Institutional Review Board due to the study's retrospective design.” On page 7, lines 208-210.

Reviewer #3: There are, however, minor residual issues that should be addressed before acceptance. The legend of Table 3 appears imprecise, as it describes ICER calculation specifically for universal screening versus no screening, while also presenting results for the targeted screening strategy. The wording should be revised to clarify that ICERs are calculated relative to the same comparator for each strategy. Additionally, there is a minor inconsistency in the reporting of utility values for hypothyroidism between the Methods and Results sections, which should be harmonized for clarity.

COMMENT 1 — Table 3 legend unclear

Response: We thank the reviewer for this helpful comment. We have revised the legend of Table 3 to clarify that ICERs for both targeted and universal screening strategies are calculated relative to the same comparator, namely, no routine screening (symptom-triggered testing).

This revision ensures clarity and consistency in the interpretation of cost-effectiveness results.

We Replace:

“ICER is calculated … for universal screening…”

with:

“Incremental cost-effectiveness ratios (ICERs) are calculated relative to no routine screening (symptom-triggered testing) for each strategy,” in Table 3 legend.

COMMENT 2 — Utility inconsistency (hypothyroidism)

We thank the reviewer for identifying this inconsistency. We have reviewed and harmonized the reporting of utility values for hypothyroidism across the Methods and Results sections to ensure consistency.

The corrected values are now consistently presented throughout the manuscript.

We keep the Utility Inputs for treated hypothyroidism at 0.94 throughout the manuscript. On page 8, line 242 of the result section of the manuscript.

We believe that these revisions have addressed all remaining reviewer concerns and have further improved the clarity, consistency, and interpretability of the manuscript. We hope that the revised version is now suitable for acceptance.

7. PLOS authors have the option to publish the peer review history of their article ([what does this mean?](https://track.editorialmanager.com/CL0/https:%2F%2Fjournals.plos.org%2Fplosone%2Fs%2Feditorial-and-peer-review-process%23loc-peer-review-history/1/010f019dcd0cc8ba-300440b9-49f3-4912-8ed4-f7e00177051b-000000/cA83hGQiN2pOzUuMyXQz8gTVMUR-B13UUgOOrMk9Sow=258)). If published, this will include your full peer review and any attached files.


If you choose “no”, your identity will remain anonymous, but your review may still be made public.


**Do you want your identity to be public for this peer review?** For information about this choice, including consent withdrawal, please see our [Privacy Policy](https://track.editorialmanager.com/CL0/https:%2F%2Fwww.plos.org%2Fprivacy-policy/1/010f019dcd0cc8ba-300440b9-49f3-4912-8ed4-f7e00177051b-000000/lB2MYvAA03JjbCC5k_C5jNgfIECykSWdcOyPzdBh5Lw=258).

Reviewer #1: **Yes:** Rashmi Paudel

Reviewer #2: **Yes:** Robert J. Chen, MD, MPH

Reviewer #3: **Yes:** Luca Bertolaccini

[NOTE: If reviewer comments were submitted as an attachment file, they will be attached to this email and accessible via the submission site. Please log into your account, locate the manuscript record, and check for the action link "View Attachments". If this link does not appear, there are no attachment files.]

To ensure your figures meet our technical requirements, please review our figure guidelines: <https://journals.plos.org/plosone/s/figures>

You may also use PLOS’s free figure tool, NAAS, to help you prepare publication quality figures: <https://journals.plos.org/plosone/s/figures#loc-tools-for-figure-preparation>.

NAAS will assess whether your figures meet our technical requirements by comparing each figure against our figure specifications.
