# Supplementary material for: A preliminary cost-utility analysis of routine myasthenia gravis and thyroid dysfunction screening in acquired comitant Esotropia
Source: PLoS One. 2026 May 28;21(5):e0350280. doi: 10.1371/journal.pone.0350280 (PMC13218454; doi:10.1371/journal.pone.0350280)
Supplement: Attachment — Submitted filename: Response_to_Reviewers_auresp_3.docx [file pone.0350280.s013.docx]

**Response to reviewers’ comments**

Reviewers' comments:

Reviewer's Responses to Questions

**Comments to the Author**

1. If the authors have adequately addressed your comments raised in a previous round of review and you feel that this manuscript is now acceptable for publication, you may indicate that here to bypass the “Comments to the Author” section, enter your conflict of interest statement in the “Confidential to Editor” section, and submit your "Accept" recommendation.

Reviewer #1: All comments have been addressed

Reviewer #2: (No Response)

Reviewer #3: (No Response)

2. Is the manuscript technically sound, and do the data support the conclusions?

The manuscript must describe a technically sound piece of scientific research with data that supports the conclusions. Experiments must have been conducted rigorously, with appropriate controls, replication, and sample sizes. The conclusions must be drawn appropriately based on the data presented.

Reviewer #1: Yes

Reviewer #2: Partly

Reviewer #3: (No Response)

3. Has the statistical analysis been performed appropriately and rigorously?

Reviewer #1: Yes

Reviewer #2: Yes

Reviewer #3: (No Response)

4. Have the authors made all data underlying the findings in their manuscript fully available?

The [PLOS Data policy](http://track.editorialmanager.com/CL0/http:%2F%2Fwww.plosone.org%2Fstatic%2Fpolicies.action%23sharing/1/010f019e14b8d58c-4fc7bb2d-dc6f-4947-90ea-23faf293c755-000000/PW5Op5oZzbMFiTZSsob-Z6PEeFQazwwxRv-Bvpcjn_Q=258) requires authors to make all data underlying the findings described in their manuscript fully available without restriction, with rare exception (please refer to the Data Availability Statement in the manuscript PDF file). The data should be provided as part of the manuscript or its supporting information, or deposited to a public repository. For example, in addition to summary statistics, the data points behind means, medians and variance measures should be available. If there are restrictions on publicly sharing data—e.g. participant privacy or use of data from a third party—those must be specified.

Reviewer #1: Yes

Reviewer #2: Yes

Reviewer #3: (No Response)

5. Is the manuscript presented in an intelligible fashion and written in standard English?

PLOS ONE does not copyedit accepted manuscripts, so the language in submitted articles must be clear, correct, and unambiguous. Any typographical or grammatical errors should be corrected at revision, so please note any specific errors here.

Reviewer #1: Yes

Reviewer #2: No

Reviewer #3: (No Response)

6. Review Comments to the Author

Please use the space provided to explain your answers to the questions above. You may also include additional comments for the author, including concerns about dual publication, research ethics, or publication ethics. (Please upload your review as an attachment if it exceeds 20,000 characters)

Reviewer #1: I reviewed the revised manuscript and found that the authors have adequately addressed the comments and suggestions raised during the previous round of review.

Reviewer #2: This revised cost–utility analysis is substantially improved, with clearer comparator definition and better alignment between base-case and sensitivity analyses. The modeling framework (decision tree + Markov) is appropriate; however, key structural limitations remain. The “targeted screening” arm is not a true risk-stratified strategy but a proportional cost-reduction scenario without differential prevalence or test performance, limiting interpretability and external validity. This should be reframed more explicitly in Methods, not only Discussion. Absolute QALY magnitudes remain inflated at the cohort level and, while clarified, still risk misinterpretation; presenting per-patient QALYs alongside cohort totals would improve transparency. The small event count (3 MG, 1 thyroid) introduces instability in prevalence-driven ICER estimates, and probabilistic sensitivity analysis cannot fully compensate for structural uncertainty. Exclusion of implementation costs biases toward screening and should be emphasized as a directional limitation.
Clarify targeted strategy definition in Methods; report per-patient QALYs; explicitly emphasize structural uncertainty and small-event limitations; tighten language.

Response:

We sincerely thank the reviewer for the thoughtful feedback and for recognizing the substantial improvements made in the revised manuscript.

In response to these final comments, we made several targeted revisions to further improve clarity and transparency.

First, we strengthened the description of the targeted screening strategy in the Methods section by explicitly stating that this approach represents a proportional reduction in testing volume rather than a formally risk-enriched screening strategy (on page 4, lines 117-119). This clarification was previously emphasized in the Discussion and has now been incorporated earlier in the manuscript.

Second, to improve transparency regarding QALY interpretation, we now report per-patient QALY estimates alongside cohort-level totals in the Results section (on page 8, lines 253-256 and page 9, lines 267-268 and table 2 legends). This helps avoid misinterpretation of large absolute cohort QALY values.

Third, we further strengthened the limitations section by explicitly stating that probabilistic sensitivity analysis addresses parameter uncertainty but cannot fully resolve structural uncertainty related to model assumptions and the small number of observed disease events (on page 15, lines 489-491).

Fourth, we revised the limitations section to more explicitly state that exclusion of implementation costs likely biases the model in favor of screening and may underestimate the full economic burden of universal implementation (on page 15, lines 497-499).

Finally, we made minor language refinements throughout the manuscript for clarity and corrected minor wording inconsistencies (on page 13, lines 399-401).

We appreciate the reviewer’s thoughtful comments, which have helped further strengthen the manuscript.

Reviewer #3: (No Response)

7. PLOS authors have the option to publish the peer review history of their article ([what does this mean?](https://track.editorialmanager.com/CL0/https:%2F%2Fjournals.plos.org%2Fplosone%2Fs%2Feditorial-and-peer-review-process%23loc-peer-review-history/1/010f019e14b8d58c-4fc7bb2d-dc6f-4947-90ea-23faf293c755-000000/J7B3PPXuVYhAPOzTQkqDx1gN2crg5_vKIEKTDNdk44I=258)). If published, this will include your full peer review and any attached files.


If you choose “no”, your identity will remain anonymous but your review may still be made public.


**Do you want your identity to be public for this peer review?** For information about this choice, including consent withdrawal, please see our [Privacy Policy](https://track.editorialmanager.com/CL0/https:%2F%2Fwww.plos.org%2Fprivacy-policy/1/010f019e14b8d58c-4fc7bb2d-dc6f-4947-90ea-23faf293c755-000000/qjMjnfaMmX4QxTqIccN_24HHCzlCPNSaQquk7c52MUM=258).

Reviewer #1: **Yes:** Rashmi Paudel

Reviewer #2: **Yes:** Robert J. Chen, MD

Reviewer #3: **Yes:** Luca Bertolaccini

[NOTE: If reviewer comments were submitted as an attachment file, they will be attached to this email and accessible via the submission site. Please log into your account, locate the manuscript record, and check for the action link "View Attachments". If this link does not appear, there are no attachment files.]

To ensure your figures meet our technical requirements, please review our figure guidelines: <https://journals.plos.org/plosone/s/figures>

You may also use PLOS’s free figure tool, NAAS, to help you prepare publication quality figures: <https://journals.plos.org/plosone/s/figures#loc-tools-for-figure-preparation>.

NAAS will assess whether your figures meet our technical requirements by comparing each figure against our figure specifications.
